# A gene expression map of host immune response in human brucellosis

**DOI:** 10.3389/fimmu.2022.951232

**Published:** 2022-08-01

**Authors:** Ioannis Mitroulis, Akrivi Chrysanthopoulou, Georgios Divolis, Charalampos Ioannidis, Maria Ntinopoulou, Athanasios Tasis, Theocharis Konstantinidis, Christina Antoniadou, Natalia Soteriou, George Lallas, Stella Mitka, Mathias Lesche, Andreas Dahl, Stephanie Gembardt, Maria Panopoulou, Paschalis Sideras, Ben Wielockx, Ünal Coskun, Konstantinos Ritis, Panagiotis Skendros

**Affiliations:** ^1^ Laboratory of Molecular Hematology, Democritus University of Thrace, University Hospital of Alexandroupolis, Alexandroupolis, Greece; ^2^ First Department of Internal Medicine, Democritus University of Thrace, University Hospital of Alexandroupolis, Alexandroupolis, Greece; ^3^ Department of Biological Applications and Technology, University of Ioannina, Ioannina, Greece; ^4^ Biomedical Research Foundation Academy of Athens, Center for Clinical, Experimental Surgery and Translational Research, Athens, Greece; ^5^ Institute for Clinical Chemistry and Laboratory Medicine, Faculty of Medicine, Technische Universität Dresden, Dresden, Germany; ^6^ Laboratory of Microbiology, Democritus University of Thrace, University Hospital of Alexandroupolis, Alexandroupolis, Greece; ^7^ R&D Department, P. Zafiropoulos S.A., Athens, Greece; ^8^ School of Biomedical Sciences, International Hellenic University, Thessaloniki, Greece; ^9^ DRESDEN-concept Genome Center, Center for Molecular and Cellular Bioengineering, Technische Universität Dresden, Dresden, Germany

**Keywords:** brucellosis, immunity, transcriptomics, macrophages, polymorphonuclear neutrophils, peripheral blood mononuclear cells

## Abstract

Brucellosis is a common zoonotic disease caused by intracellular pathogens of the genus *Brucella*. *Brucella* infects macrophages and evades clearance mechanisms, thus resulting in chronic parasitism. Herein, we studied the molecular changes that take place in human brucellosis both *in vitro* and *ex vivo.* RNA sequencing was performed in primary human macrophages (Mφ) and polymorphonuclear neutrophils (PMNs) infected with a clinical strain of *Brucella* spp. We observed a downregulation in the expression of genes involved in host response, such as TNF signaling, IL-1β production, and phagosome formation in Mφ, and phosphatidylinositol signaling and TNF signaling in PMNs, being in line with the ability of the pathogen to survive within phagocytes. Further transcriptomic analysis of isolated peripheral blood mononuclear cells (PBMCs) and PMNs from patients with acute brucellosis before treatment initiation and after successful treatment revealed a positive correlation of the molecular signature of active disease with pathways associated with response to interferons (IFN). We identified 24 common genes that were significantly altered in both PMNs and PBMCs, including genes involved in IFN signaling that were downregulated after treatment in both cell populations, and *IL1R1* that was upregulated. The concentration of several inflammatory mediators was measured in the serum of these patients, and levels of IFN-γ, IL-1β and IL-6 were found significantly increased before the treatment of acute brucellosis. An independent cohort of patients with chronic brucellosis also revealed increased levels of IFN-γ during relapse compared to remissions. Taken together, this study provides for the first time an in-depth analysis of the transcriptomic alterations that take place in human phagocytes upon infection, and in peripheral blood immune populations during active disease.

## Introduction

Brucellosis is a common bacterial zoonotic disease worldwide and an emerging zoonosis in several developed countries (1, 2). Despite its importance in public health brucellosis remains widespread and neglected in many areas, including southeastern Europe, Asia, Central and Latin America, and Africa (2, 3). It is caused by various species of the bacterial genus *Brucella*, which mainly infect domestic animals, especially goats, sheep, and cows, and use them as natural reservoirs. The disease is transmitted to humans by consumption of unpasteurized milk and dairy products or by occupational contact with infected animals. Additionally, *Brucella* is highly infectious through the aerosol route, thus is considered as one of the most common laboratory-acquired pathogens and is also classified as a category B agent on the biodefense list (4).

Human brucellosis causes high morbidity and protean clinical manifestations, mimicking many infectious and non-infectious diseases since it can affect multiple organs. Despite early diagnosis and prolonged therapy with antibiotics is associated with substantial residual disability (4). Up to 30% of patients develop chronic disease, which is characterized by atypical clinical manifestations, high frequency of focal complications such as spondylitis, chronic fatigue syndrome, and relapses (4, 5).

Host protection against *Brucella* and prevention of its intracellular parasitism in macrophages depends on cell-mediated immunity, involving adequate Th1 immune response, with significant production of interferon-gamma (IFN-γ) (5). Previous data support also a key role of innate immunity and neutrophils in early proinflammatory responses against *Brucella* that may affect T-cell dynamics during infection (5–7). On the other hand, *Brucella* has developed various stealthy strategies to evade innate and adaptive immune responses, in order to establish intracellular long-term survival and replication (8, 9). Several studies have demonstrated that patients with chronic brucellosis display defective cell-mediated immunity (brucellosis-acquired cellular anergy) probably due to modulation of host cellular immunity by *Brucella* (5). However, immunopathogenesis of human brucellosis remains incompletely understood and integrated molecular data that characterize complex interactions between *Brucella* and host immunity are missing today.

Here, we shed light on the transcriptomic alterations that macrophages (Mφ) and polymorphonuclear neutrophils (PMNs) undergo during the crucial early events of *Brucella* infection. Moreover, we analyze the transcriptomic alterations that take place concomitantly in peripheral blood mononuclear cells (PBMCs) and PMNs of patients upon treatment, uncovering candidate molecular targets and pathways that may characterize active infection and disease eradication.

## Materials and methods

### Patients

Ten adult patients with acute brucellosis were recruited. EDTA anticoagulated blood and serum were collected from patients with active brucellosis before the initiation of antibiotic treatment and three months after the completion of treatment, when all patients were successfully treated. The diagnosis was based on compatible clinical manifestations in combination with high serum titers of anti-*Brucella* antibodies (Wright’s agglutination test ≥160) or a four-fold increase of the initial titers in two-paired samples drawn 2 weeks apart, or/and Brucella isolation, according to Centers for Disease Control and Prevention (CDC)/Council of State and Territorial Epidemiologists (CSTE) Laboratory Criteria for Diagnosis (10). None of these patients suffered any relapse during a six-month post-treatment follow-up period. Patient characteristics and treatment are described in **Table 1**. PBMCs and PMNs were simultaneously isolated from patients. PBMCs and PMNs were also isolated from ten healthy, sex and age-matched, subjects who served as controls (**Table 2**). Sera from a second cohort of 25 chronic relapsing brucellosis patients at clinical relapse and remission, were also used. These patients had a disease duration of ≥12 months in combination with positive serum agglutination tests (SATs) or/and complement fixation test, or/and Brucella isolation (**Supplementary Table S1**).

**Table 1:** Characteristics of patients with acute brucellosis (AB).

Patient#	Sex	Age(years)	Symptoms/Findings	Route of transmission	Wright SAT	Bloodculture	Antibiotictreatment
AB1	F	40	Fatigue, malaise myalgias, arthralgias	Consumption	1/640	N/A	Rifampicin Doxycycline
AB2	F	53	Fever, sweating, arthralgias, peripheral arthritis	Consumption	1/320	Negative	Rifampicin Doxycycline Amikacin
AB3	M	31	Fever, sweating, fatigue	Consumption/contact	1/320	Negative	Rifampicin Doxycycline Amikacin
AB4	M	36	Fever, sweating, malaise, fatigue	Consumption	1/5120	Negative	Rifampicin Doxycycline Amikacin
AB5	M	55	Fever, sweating, lumbar spondylitis	Contact	1/160	*Brucella spp*	Rifampicin Doxycycline Amikacin
AB6	M	39	Fever, myalgia	Contact	1/320	*Brucella spp*	Rifampicin Doxycycline Amikacin
AB7	M	64	Sweating, fatigue, low back pain	Consumption	1/320	*Brucella spp*	Rifampicin Doxycycline Amikacin
AB8	F	45	Fatigue, lumbar spondylitis	Consumption	1/640	N/A	Rifampicin Doxycycline Amikacin
AB9	M	18	Fever, sweating, malaise, fatigue myalgias, arthralgias	Consumption/contact	1/160	*Brucella spp*	Rifampicin Doxycycline Amikacin
AB10	M	52	Fatigue, myalgias, arthralgias, peripheral arthritis	Contact/REV1 vaccine	1/160	Negative	Rifampicin Doxycycline
Age (years, mean ± SD)		43.3 ± 13.4					

F, female; M, male; N/A, not available; SAT, serum agglutination test; SD, standard deviation.

Duration of antibiotic treatment was 8-12 weeks for rifampicin (600 mg/daily) and doxycycline (200 mg/daily), and 2-3 weeks for Amikacin (1 gr/daily).

**Table 2:** Demographic characteristics of healthy subjects (controls).

Control#	Sex	Age (years)
C1*	F	38
C2*	M	47
C3*	M	35
C4*	M	34
C5^	M	55
C6^	M	40
C7^	F	51
C8^	F	44
C9^	M	23
C10^	M	52
Age (years, mean ± SD)		41.9 ± 9.8

F, female; M, male; SD, standard deviation. All controls had no previous history of brucellosis and yielded a negative Wright serum agglutination test (<1/80). *Isolation of PBMCs that were used for macrophage differentiation and *in vitro* infection with *Brucella spp*, ^isolation of PMNs that were used for *in vitro* infection with *Brucella spp*.

Exclusion criteria were co-existence of other infectious, neoplastic or autoimmune disease, administration of immunomodulating agents or vaccination for at least 4 weeks before the entry to study, and pregnancy. The study was approved by the Local Scientific and Ethics Committee of the University Hospital of Alexandroupolis, Greece (Approval Number #1195/19-12-2017). All subjects provided written informed consent in accordance with the principles expressed in the Declaration of Helsinki.

### PBMCs and PMNs isolation

PBMCs and PMNs were isolated from EDTA blood by Histopaque (Sigma-Aldrich, 1077 and 1119) double-gradient density centrifugation (30 minutes, 700g, at 20°C-25°C) according to the manufacturer’s recommendations. Then, cells were washed once with phosphate buffered saline (PBS-1x, ThermoFisher Scientific) and cultured. Cell purity was ≥ 98% as assessed by microscopy (May Grunwald-Giemsa staining) and/or flow cytometry.

For RNA experiments, cell pellet was resuspended in 1mL TRIzol reagent (ThermoFisher Scientific) and the extraction procedure was performed immediately after cell isolation, according to the manufacturer’s instructions.

### Mφ differentiation

Human Mφ were differentiated from isolated PBMCs from four controls (**Table 2**). To promote Mφ differentiation, monocytes were isolated in RPMI-1640 (ThermoFisher Scientific) using plastic adherence. Non-adherent cells were removed after 6h (day 0). Adherent cells were cultured in RPMI-1640 culture medium supplemented with 10% autologous serum for 6 additional days (day 1-6) and penicillin/streptomycin solution (ThermoFisher Scientific) (11). Cell cultures were washed with prewarmed PBS-1x and culture medium was changed every other day, to ensure the removal of remaining contaminating lymphocytes. On day 7, cell culture medium was removed and *in vitro* infection with *Brucella* was performed.

### Phenotypic characterization of Mφ

To assess the differentiation status of human macrophages, fixation and permeabilization were performed with 4% paraformaldehyde and Triton-X (Sigma-Aldrich), respectively. Then, cells were stained using a mouse monoclonal anti-CD68 antibody (Clone: KP1, ThermoFisher Scientific) for 1 hour. A rabbit-anti mouse IgG Alexa Fluor 594 (ThermoFisher Scientific) was used as secondary antibody. DAPI solution (Ibidi) was used as nuclear counterstain. Samples were visualized with a fluorescence microscope (OLYMPUS BX51) with a fixed Nikon camera (model DS-Fi1, lens 100x) (**Supplementary Figure S1A**).

### *In vitro* infection

A clinical strain of *Brucella* spp., isolated from peripheral blood from a patient with acute brucellosis, was used for *in vitro* experiments. Isolate was presumptively identified as *B. melitensis* by automated system VITEK 2 (bioMérieux), based on the biochemical characteristics of isolate. The isolate was aliquoted, and stored at −70°C until used. Bacterial inoculum for cell infection was cultured on blood agar for 3 days under aerobic conditions, at 37°C and 5% CO_2_ according to the literature and American Society for Microbiology (ASM) guidelines (12, 13). Bacterial suspension with 0.5 McFarland was opsonized for 30 minutes using human serum and then diluted in RPMI and ~ 10^7^ bacteria in 0.5 ml of RPMI were added to each well (20 MOI) of PMNs or Mφ. Subsequently, cells were cultured for 0.5h for PMNs and 2h and 24h for Mφ. After a washing step with PBS, cells were resuspended in TRIzol reagent (ThermoFisher Scientific) and the RNA extraction procedure was performed immediately, according to the manufacturer’s instructions. Untreated PMNs and untreated Mφ, cultured for 0.5h or 2h respectively, served as control. The experimental procedure with *Brucella* spp. was performed at biosafety level 3. The above time points and concentrations were optimal for Mφ or PMNs stimulation, and established in preliminary experiments.

### Assessment of phagocytosis in Mφ and PMNs

To evaluate phagocytosis in Mφ and PMNs, cells were fixed with 4% paraformaldehyde (Sigma-Aldrich), permed with Triton-X (Sigma-Aldrich) and then stained using a mouse monoclonal anti-Brucella antibody (LSBio) for 1 hour. After thorough washes with PBS-1x, a rabbit-anti mouse IgG Alexa Fluor 594 (ThermoFisher Scientific) was used as secondary antibody. DAPI solution (Ibidi) was used as nuclear counterstain. Samples were visualized with either a fluorescence microscope (OLYMPUS BX51) with a fixed Nikon camera (model DS-Fi1, lens 40x or 60x) or a confocal microscope (Spinning Disk Andor Revolution Confocal System, Ireland) with PLAPON 606O/TIRFM-SP, NA 1.45 and UPLSAPO 100XO, NA 1.4 objectives (Olympus) (**Supplementary Figures S1B, C**).

To further evaluate phagocytosis in PMNs, cells were analyzed by flow cytometry, using the neutrophil-specific marker CD66b (PerCP-Cyanine5.5 conjugated CD66b, Biolegend). Bacteria were stained using a mouse monoclonal anti-Brucella antibody (LSBio), detected with a rabbit anti-mouse Alexa Fluor 647 (ThermoFisher Scientific) (**Supplementary Figure S1D**).

### RNA sequencing

RNA sequencing for Mφ and PBMCs was performed as previously described (14). To analyze RNA sequencing data, fragments were aligned with GSNAP (2020–12–16) to the Homo sapiens (human) genome assembly GRCh38 (hg38) from Genome Reference Consortium, and Ensembl annotation version 98 was used for the splice site support. Uniquely aligned fragments were counted with featureCounts (subread v2.0.1), again with the support of the Ensembl annotation. The exploratory analysis was performed with the DESeq 2 (v1.24.0) package within R (v3.6.3). Bias for patients was assessed using an exploratory correction with the variance stabilized transformation data of DESeq2 and the removeBatchEffect function of edgeR (3.26.8). Differential expression between before and after treatment was performed with a correction for patient.

For PMNs, 1000 ng of total RNA were used for the preparation of cDNA libraries, using the TruSeq RNA Library Preparation Kit v2 (Illumina), according to the manufacturer’s instructions. Library quality was evaluated using the Agilent DNA 1000 Kit (Agilent) with an Agilent 2100 Bioanalyzer. Quantification was performed by amplifying a set of six pre-diluted DNA standards (KAPA Biosystems) and diluted cDNA libraries by RT-qPCR. Isomolar quantities of up to 20 cDNA libraries, barcoded with different adaptors, were multiplexed. Sequencing was performed in a single-end manner at the Greek Genome Center, using a NextSeq 500/550 75c kit (Illumina) for the *in vitro* samples and a NovaSeq 6000 SP 100c kit (Illumina) for the *ex vivo* samples, generating 75 bp and 100 bp long reads, respectively, and an average of 25 million reads per library. Raw sequence data in FastQ format were uploaded to the Galaxy web platform, and standard tools of the public server “usegalaxy.org” were used for subsequent analysis (15). Briefly, quality control of raw reads was performed with FastQC (v072+galaxy1), followed by the removal of adapter sequences and low-quality bases using Trim Galore! (v0.6.3). Next, HISAT2 (v2.2.1+galaxy0) was applied for the alignment of trimmed reads to the Homo sapiens genome assembly GRCh37 (hg19) from Genome Reference Consortium. Assessment of uniform read coverage for exclusion of 5’/3’ bias and evaluation of RNA integrity at the transcript level were performed using Gene Body Coverage (v2.6.4.3) and Transcript Integrity Number (v2.6.4.1) tools, respectively. Differential gene expression was determined with DESeq2 (v2.11.40.6+galaxy1), using the count tables generated from HTSeq-count (v0.9.1) as input. The variability within and between individuals in this paired-data study was incorporated in the analysis, considering the treatment as the primary factor and the individual/patient as the secondary factor affecting gene expression. RNA sequencing data are provided in **Supplementary File S1**.

Pathway and biological processes analysis was performed using the Enrichr analysis tool (14, 16). Heat maps were generated using the Morpheus software, https://software.broadinstitute.org/morpheus (Broad Institute). Gene set enrichment (GSEA) pre-ranked analysis (1000 permutations, minimum term size of 15, maximum term size of 500) was performed using the GSEA software (Broad Institute). Gene sets were ranked by taking the -log10 transform of the p-value and signed as positive or negative based on the direction of fold change. Annotated gene sets from Molecular Signatures Database (MSigDB) were used as input (16).

### Cytokine measurement

The levels of cytokines were measured using the LEGENDplex™ Multi-Analyte Flow Assay Kit (Biolegend) in a CyFlow Cube 8 flow cytometer (Sysmex Partec, Germany), according to the manufacturer’s instructions. For comparisons between the groups the Wilcoxon signed-rank test for paired samples was used. Statistical analysis was performed using GraphPad Prism (version 9.0, GraphPad Inc., La Jolla, CA). Significance was set at p < 0.05.

## Results

### Analysis of the molecular signature of human macrophages infected *in vitro* with *Brucella* spp.

**Figure 1:**
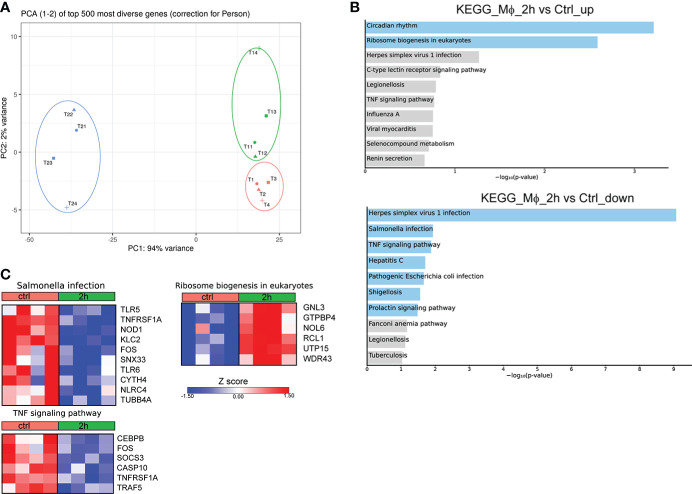
Alterations in the transcriptomic profile of human Mφ infected *in vitro* for 2h with *Brucella* spp.**(A)** Principal component analysis (PCA) of the transcriptome of all 12 Mφ samples. T1-T4 represent untreated control Mφ, T11-T14 represent samples from Mφ at 2h post-infection and T21-T24 represent samples at the 24h time point. **(B)** Pathway analysis of the DEGs at 2h post-infection compared to control, using the KEGG database as reference. Light blue color represents statistical significance **(C)** Heatmaps depicting the DEGs of the respective pathways.

To provide a time-course analysis of the molecular alterations of human Mφ during infection with *Brucella* spp., we performed *in vitro* infection of human Mφ, derived from the differentiation of peripheral blood monocytes from control subjects, and compared the transcriptomic signature of untreated Mφ compared to that of infected cells at 2h and 24h post-infection. Principal component analysis (PCA) revealed that there was a prominent change in the transcriptomic profile of Mφ at 24h after infection compared to untreated cells and cells at 2h after infection (**Figure 1A**). Pathway analysis, using the Kyoto Encyclopedia of Genes and Genomes (KEGG) database, of the significantly upregulated differentially expressed genes (DEG) (False Discovery Rate/FDR <0.01) between untreated Mφ and Mφ at 2h post-infection revealed an overrepresentation of circadian rhythm and ribosome biogenesis pathways, whereas downregulated DEGs were enriched in pathways associated with viral infection and infection from intracellular pathogens processes, including herpes simplex virus 1 infection, hepatitis C, and *Salmonella* infection and TNF signaling (**Figures 1B, C**). Interestingly, we observed a decreased expression of genes encoding proteins critical in pathogen recognition, such as *NOD1, TLR5, TLR6* and *NLRC4* (**Figure 1C**).

**Figure 2:**
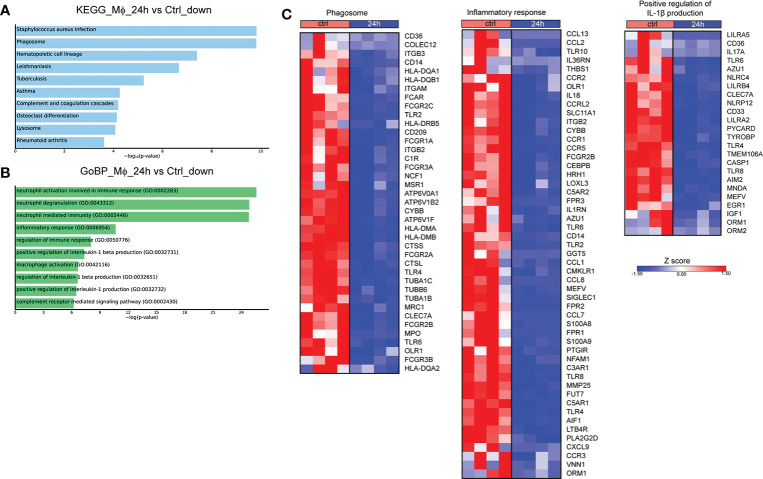
Transcriptomic profiling of human Mφ infected *in vitro* with *Brucella spp* at 24h post-infection. **(A)** Pathway analysis of the DEGs with the highest variance at 24h post infection compared to control, using the KEGG database as reference. **(B)** Enriched biological processes in which the downregulated genes are involved. **(C)** Heatmaps depicting the DEGs of the phagosome pathway, the inflammatory response and positive regulation of IL-1β production biological processes.

We next assessed the molecular changes that take place at 24h post-infection. Pathway analysis of the downregulated DEGs with the highest variance (log2 fold change > 2 and < -2, FDR<0.01) showed overrepresentation of pathways associated with infection with *S. aureus* and infection with intracellular pathogens, such as leishmaniasis and tuberculosis, as well as the pathways associated with phagosome and lysosome (**Figure 2A**). No statistically significant pathway was observed in the respective analysis of upregulated genes. Further, analysis of the DEGs that were downregulated at 24h after infection revealed that they are involved in biological processes associated with inflammation, and more specifically with the production of IL-1 and Mφ function (**Figure 2B**). Regarding the genes involved in the aforementioned pathways, there was a downregulation of several genes involved in the phagosome formation and function at 24h after infection, including those encoding for several Fcγ receptors (*FCGR1A, FCGR2A, FCGR2B, FCGR2C, FCGR3A, FCGR3B*), toll-like receptors (*TLR2, TLR4, TLR6*), other sensors of pathogen-associated molecular patterns (*CLEC7A, CD14*), integrins and other receptors involved in phagocytosis (*ITGB3, ITGAM, ITGB2, CD36*) (**Figure 2C**). We also observed a downregulation in the expression of genes encoding cytokines and cytokine receptors of the IL-1 family (*IL18*, *IL1RN, IL36RN*), chemokines (*CCL1, CCL2, CCL7, CCL8, CCL13, CXCL9*) and chemokine receptors (*CCR1, CCR2, CCR3, CCR5*), formyl peptide receptors (*FPR1, FPR2, FPR3*), and complement anaphylatoxin receptors (*C3AR1, C5AR1, C5AR2*) (**Figure 2C**). Regarding the regulation of IL-1 production, we observed the downregulation of several genes encoding inflammasome sensors (*NLRC4*, *NLRP12*, *MEFV*, *AIM2)*, the adaptor *PYCARD*, and the gene that encodes the effector *CASP1* (**Figure 2C**). Taken together, infection of Mφ with *Brucella* spp. drives major changes in the transcriptomic profile of infected Mφ, downregulating a plethora of genes involved in the formation of phagosomes and the recognition of pathogens, in an effort to preserve pathogen survival within Mφ.

### Analysis of the molecular signature of human PMNs infected *in vitro* with *Brucella* spp.

**Figure 3:**
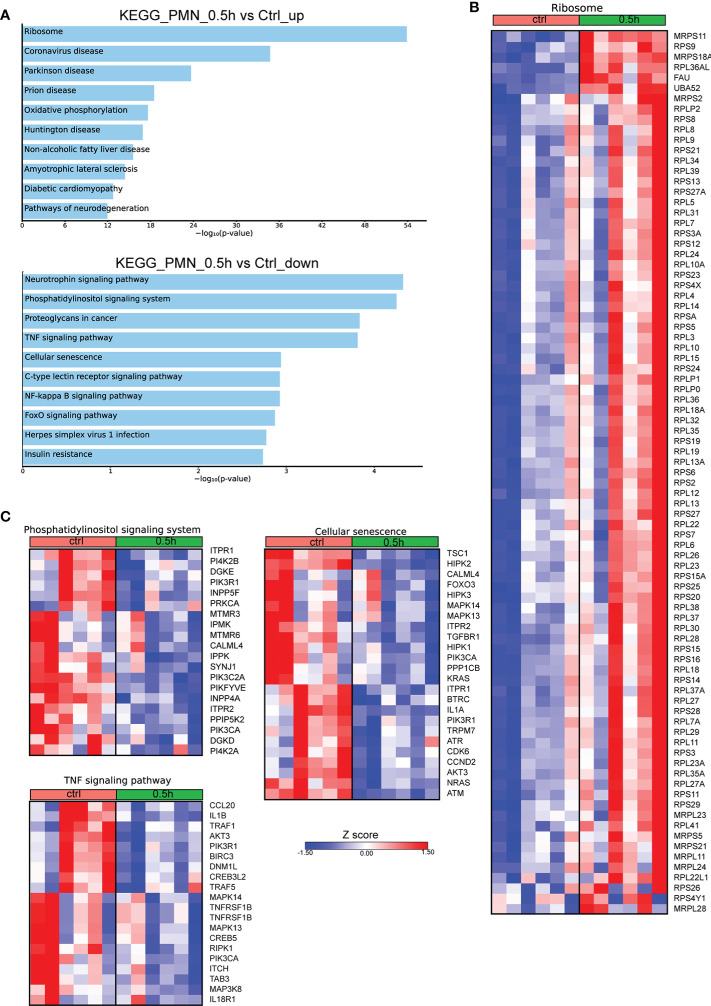
Alterations in the transcriptomic profile of human PMNs infected *in vitro* with *Brucella* spp. **(A)** Pathway analysis of the DEGs from PMNs at 0.5h post infection with *Brucella spp* compared to control, using the KEGG database as reference. **(B)** Heatmap depicting the DEGs of the ribosome pathway. **(C)** Heatmaps depicting the DEGs of the pathways enriched for downregulated genes.

Even though Mφ are the major cell population infected by *Brucella* spp, it has been previously shown that this pathogen can also infect neutrophils (7, 17). To characterize the molecular signature of infected PMNs with *Brucella spp*, we performed *in vitro* infection of human PMNs for 0.5h, derived from control subjects, and compared the transcriptomic signature of untreated PMNs to that of infected cells. Pathway analysis of the significantly overexpressed DEGs (FDR<0.01), using the KEGG database, highlighted Ribosome as the top upregulated pathway in *Brucella*-infected PMNs (**Figure 3A**). Notably, almost all genes (75 out of the 79) encoding for structural proteins of both small and large subunits of cytoplasmic ribosomes were found significantly upregulated (**Figure 3B**). Respective analysis of the downregulated DEGs demonstrated modulation of several pathways, some of which were also downregulated in *Brucella*-infected Mφ at 2h post-infection, such as TNF signaling and herpes simplex virus 1 infection (**Figures 1B**, **3C**). However, various inflammation-related biological processes were significantly downregulated selectively in PMNs, namely the phosphatidylinositol signaling, NF-kappa B signaling, and cellular senescence pathways (**Figure 3C**). Amongst the downregulated transcripts in *Brucella*-infected PMNs, we identified several modulators of apoptosis (*BIRC3*, *FOXO3, DNM1L, ITPR1, TRAF1, TRAF5*) and inflammation, as exemplified by decreased mRNA expression of cytokines and corresponding receptors of the IL-1 family (*IL1A*, *IL1B, IL18R1*), chemokines (eg. *CCL20*), and various signaling mediators, such as kinases (*AKT3, ATM, ATR, CDK6, DGKD, DGKE, IPMK, IPPK, MAPK13, MAPK14*, *RPK1*) (**Figure 3C**).

### Transcriptomic profiling of active human brucellosis

**Figure 4:**
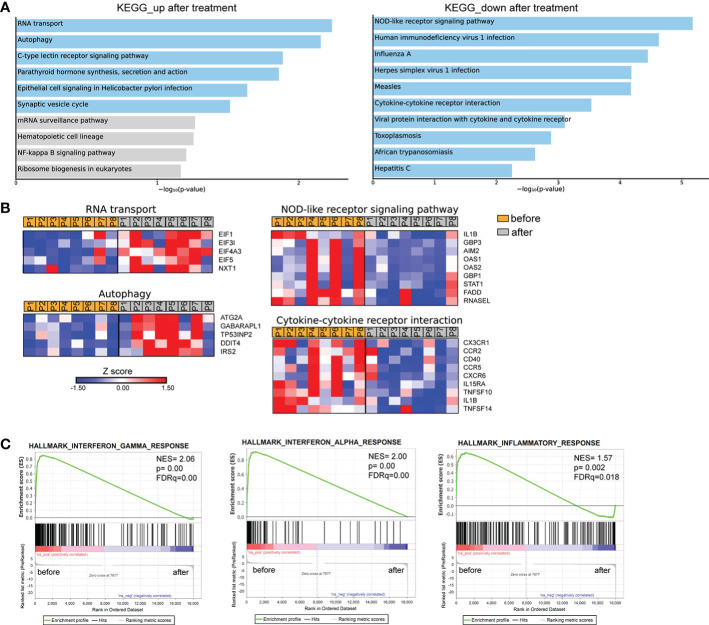
Transcriptomic analysis of PMNs from patients with brucellosis before treatment initiation and after successful completion of treatment. **(A)** Pathway analysis of the DEGs from PMNs after treatment compared to PMNs isolated from the same patients (paired-data analysis) during active brucellosis, using the KEGG database as reference. Light blue color represents statistical significance **(B)** Heatmaps depicting the DEGs of the respective pathways. P1-P8 refer to different patients. **(C)** GSEA for genes related to response to interferons, and inflammation.

We further investigated the transcriptomic signature of active human brucellosis. To do so, PMNs were isolated from eight patients with active brucellosis before the initiation of antibiotic treatment (active disease) and three months after completion of the antibiotic treatment, when patients were free of symptoms (remission). Transcriptomic analysis identified 318 DEGs (FDR<0.1). DEGs that were upregulated after treatment are involved in RNA transport and autophagy pathways, whereas downregulated DEGs after treatment are involved in NOD-like receptor signaling pathway and cytokine-cytokine receptor interaction pathways, as well as several pathways associated with infectious diseases (**Figure 4A**). The upregulated genes that encode proteins involved in RNA transport were the members of the eukaryotic initiator factors (EIF) family *EIF1, EIF3I, EIF4A3, EIF5*, and the genes of the autophagy pathway were *ATG2A, GABARAPL1, TP53INP2, DDIT4* and *IRS2* (**Figure 4B**). On the other hand, we observed a downregulation of critical genes in immune regulation, such as *IL1B, CX3CR1, CCR2, CCR5, CXCR6, STAT1, AIM2*, and *CD40*, as well as genes associated with interferon signaling, such as *OAS1, OAS2, GBP1* and *GPB3* (**Figure 4B**). We further performed gene set enrichment analysis (GSEA) using the Hallmark Gene Set collection of the Molecular Signatures Database. We observed a positive correlation of the transcriptomic signature of PMNs during active brucellosis with IFN-γ and IFN-α response and with inflammatory response (**Figure 4C**). Moreover, comparing the transcriptomic profiling of *ex vivo* PMNs after successful completion of treatment versus that of *in vitro Brucella*-infected PMNs, we found that 188 genes (59% of the *ex vivo* identified DEGS) were commonly regulated in both datasets (**Supplementary Figure S2A**). Furthermore, the majority of commonly regulated genes (111 out of 188) followed a reverse pattern of differential expression (eg. upregulated upon *in vitro Brucella* infection and downregulated *ex vivo*, upon successful completion of treatment, **Supplementary Figures S2B, C**).

**Figure 5:**
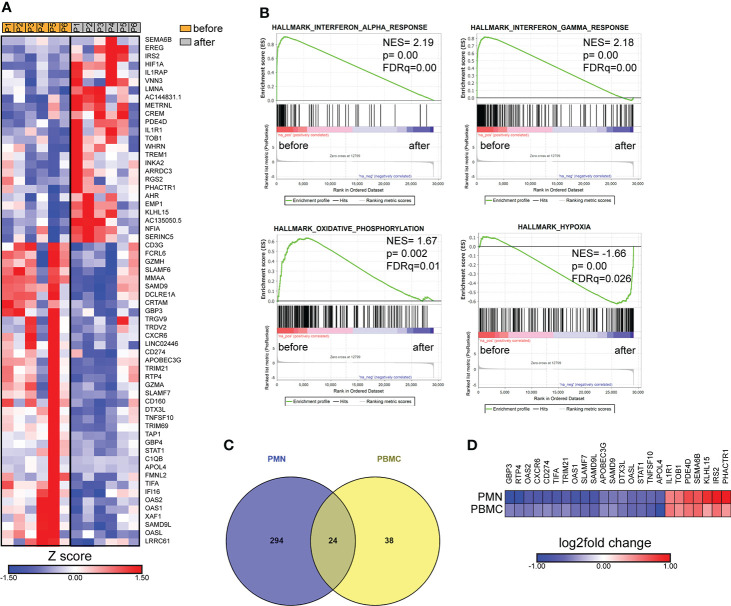
Transcriptomic analysis of PBMCs from patients with brucellosis before treatment initiation and after successful completion of treatment. **(A)** Heatmap depicting the DEGs from PBMCs from patients with acute brucellosis before treatment initiation and from the same patients (paired analysis) after successful treatment. P1-P6 refer to different patients. **(B)** GSEA for genes related to response to interferons, oxidative phosphorylation and hypoxia. **(C)** Venn diagram and **(D)** heatmap depicting the common genes that were significantly differentially expressed in PMNs and PBMCs from patients with brucellosis after treatment.

In parallel, we performed transcriptomic analysis of PBMCs isolated from six patients with active brucellosis before and after antibiotic treatment. Transcriptomic analysis identified 62 genes with significantly altered expression (FDR<0.1) after treatment (**Figure 5A**). We observed that successful treatment resulted in the increased expression of *HIF1A*, a critical regulator of inflammation, and of the genes that encode IL-1 receptor *IL1R1*, and its accessory protein *IL1RAP*, which form a complex that mediates IL-1 signal transduction (**Figure 5A**). On the other hand, there was a downregulation in the expression of genes that play a major role in immune function, such as *CD274*, which encodes PD-L1, *STAT1*, *CD3G*, the intracellular immunoglobulin receptor *TRIM21*, *CXCR6*, the lymphocytic activation molecules *SLMF6, SLAMF7* and genes that encode proteins important in effector cell cytolytic processes, such as *CD160*, *GZMA*, *GZMH* (**Figure 5A**). Moreover, several identified genes are involved in interferon-related activation pathways, such as *GPB3*, *GPB4*, *OAS1*, *OAS2*, *OASL*, *IFI16*, and *XAF1* (**Figure 5A**). In the same line, GSEA analysis revealed that the gene sets with the most significant positive association with active disease were IFN signaling and OXPHOS, whereas the one with the most significant negative association was the hypoxia gene set (**Figure 5B**). Notably, we further identified 24 genes that were differentially expressed both in PMNs and PBMCs (**Figure 5C**). Among these common genes, *CXCR6*, *TRIM21*, *SLAM7*, *CD274* and the genes associated with IFN signaling *OASL, OAS1, OAS2, GBP3*, and *STAT1* were downregulated in both datasets, whereas *IL1R1* was commonly upregulated (**Figure 5D**).

### Cytokine levels in acute brucellosis

**Figure 6:**
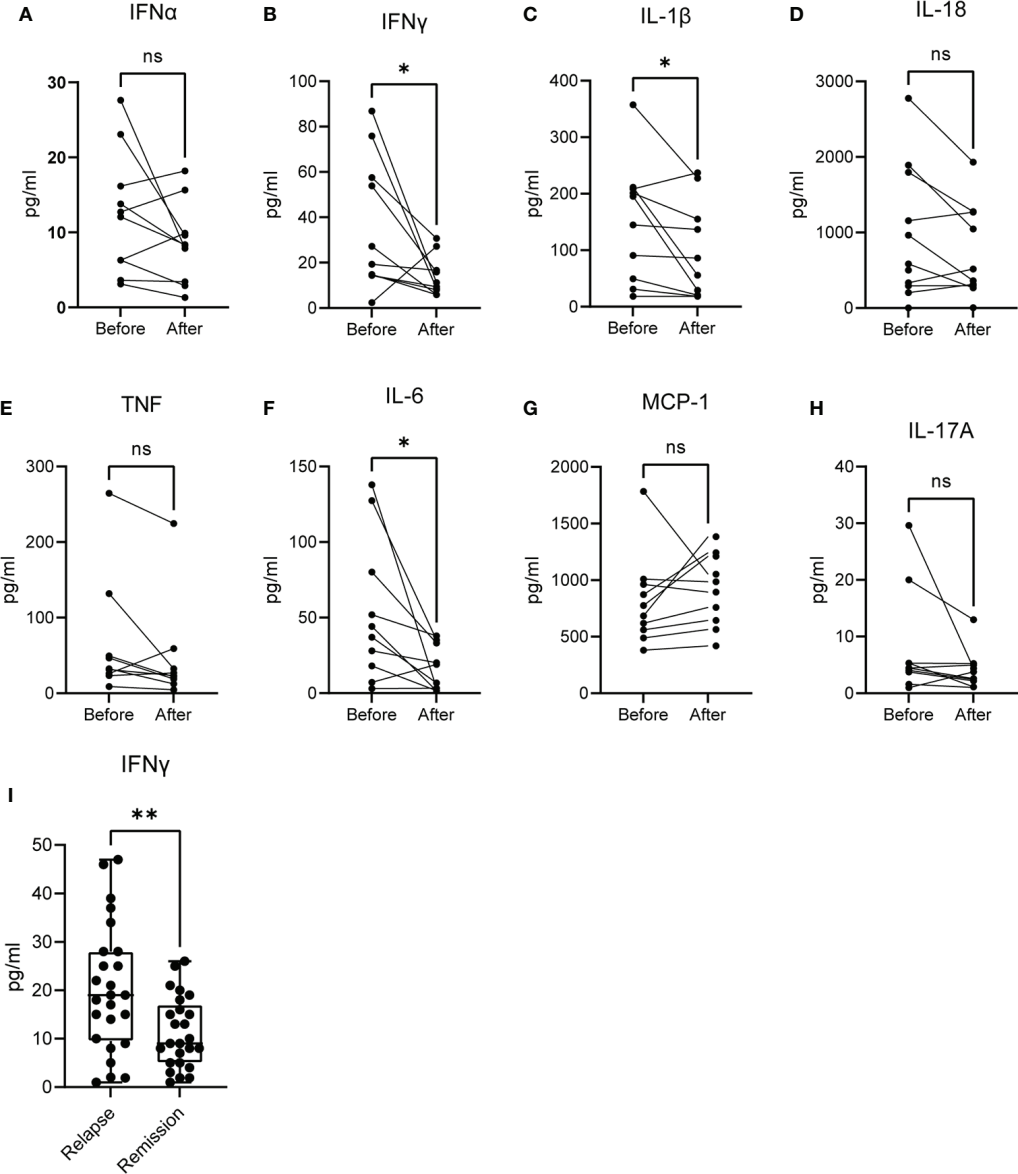
Levels of cytokines in the serum of patients with active brucellosis. **(A–H)** Levels of IFN-α, IFN-γ, IL-1β, IL-18, TNF, IL-6, MCP-1 and IL-17A in the serum of patients with acute brucellosis before treatment initiation and after successful treatment. **(I)** Levels of IFN-γ in an independent cohort of patients with chronic relapsing brucellosis during relapse and remission. *p<0.05, **p<0.01. Wilcoxon signed rank test. ns, non significant.

To this point, we observed that the molecular signature that characterizes acute brucellosis is positively correlated with those of IFN-α and IFN-γ responses. For this reason, we measured the levels of several cytokines in the sera of patients during acute brucellosis and after successful treatment. We observed a significant downregulation in the levels of IFN-γ, IL-1β and IL-6 post-treatment, whereas there was no statistically significant difference in the levels of IFN-α, IL-18, TNF, MCP-1 and IL-17A (**Figures 6A** **–H**). We further confirmed that the levels of IFN-γ are increased in active disease in a cohort of patients with chronic relapsing brucellosis. In this cohort, the levels of IFN-γ were increased during relapse compared to remission (**Figure 6I**).

## Discussion

The interaction between *Brucella* and the host immune system is critical for the development of persistent infection or infection clearance (5, 9). To date, transcriptomic data were derived from *Brucella*-infected mouse macrophages or mouse cell lines, domestic ruminants or *Brucella*-vaccinated animals (18–24). This study analyses, for the first time, the transcriptome profile, both *in vitro*, in *Brucella*-infected primary Mφ and PMNs, and *ex vivo*, in PBMCs and PMNs derived from patients with acute brucellosis before and after treatment. This provides the molecular signature that characterizes the main host cellular immune populations during their initial interplay with invading *Brucella*, and the molecular signature of different stages of the disease.

Macrophages differentiated *in vitro* from purified peripheral blood monocytes are widely used in the literature to simulate human macrophages for *in vitro* studies (11). Different isolation strategies may affect the purity and cell yield of resulting monocytes and/or monocyte-derived macrophages, as well as the monocyte subtype and the polarization status of subsequently differentiated cells. To address the transcriptomic changes that take place during Mφ infection, we engaged cell cultures of monocytes isolated with plastic adhesion, a setup that results in the generation of Mφ with inflammatory characteristics and M1 skewing (11). Although, plastic adhesion is a straightforward, uncomplicated, and low-cost isolation method, it results in lower monocyte yield compared to other immune-based methods (11). Whilst all our samples were handled similarly, we should always take into consideration the described limitations of these *in vitro* systems when forming conclusions.

Early molecular events following phagocytosis of *Brucella* by macrophages are crucial for the activation of innate immunity leading to the induction of a favorable Th1 response (5, 8, 9). Several lines of evidence indicated that *Brucella* manipulates multiple effector mechanisms in macrophages to its benefit (5, 9). In line with this, we identified that in Mφ infected *in vitro* by a clinical strain of *Brucella spp*, the expression of several genes encoding key proteins involved in the recognition of *Brucella* and in the proinflammatory response against the pathogen were markedly suppressed. These alterations may initiate as soon as 2h post-infection being more prominent at 24h post-infection. Interestingly, most downregulated DEGs related to phagosome, TNFα signaling and IL-1β production. Indeed, previous studies reported that various *Brucella* virulence factors and pathogen-associated molecular patterns (PAMPs), such as Type IV secretory system (T4SS), lipopolysaccharide (LPS) and outer membrane lipoproteins (OMPs) modify phagosome biogenesis and trafficking in macrophages to inhibit phagolysosome fusion, and develop suitable vacuolar compartments to enable intracellular replication of the microbe (5, 9). Moreover, the current study comes in agreement with previous data demonstrated that *Brucella* Omp25 protein inhibits *in vitro* the production of TNF in human Mφ and dendritic cells preventing cell maturation and antigen presentation (25–27). Furthermore, several genes encoding members of the IL-1 family (*IL18*, *IL1RN, IL36RN*) and inflammasome complexes (*NLRC4*, *NLRP12*, *MEFV*, *AIM2, PYCARD*, *CASP1)* are significantly downregulated in *Brucella*-infected Mφ. Experimental studies indicated that inflammasomes and their effectors are essential for an initial effective immune response against *Brucella* infection (28–30). On the other hand, *Brucella* can regulate canonical and non-canonical inflammasome signaling and pyroptosis in macrophages by impairing caspase-1 and caspase-4/11 activation, and IL-1β secretion (31, 32). It is intriguing that *Brucella* downregulates macrophage *MEFV* expression, the gene responsible for familial Mediterranean fever, the prototype IL-1β-mediated autoinflammatory disease (33). Mutations in the *MEFV* gene are highly prevalent in the Middle East and Mediterranean countries where brucellosis is endemic (33). Our data further support the hypothesis that *MEFV* mutations may provide an evolutionary selective advantage to confer protection against brucellosis (34).

Recently, PMNs emerge as novel players during the initial stages of innate immune response against *Brucella* infection (7). *Brucella* resists the killing mechanisms of human PMNs and induces the early death of these cells promoting their phagocytosis by Mφ, which become vehicles for bacterial dispersion within the host (35). Studies in murine brucellosis proposed that infected PMNs attenuate cellular adaptive immunity, given that depletion of PMNs favored bacterial elimination (36). Based on these, this study examined the early transcriptome alterations of *in vitro Brucella*-infected neutrophils, before their premature death. *Brucella spp*-infected PMNs were characterized by increased expression of genes associated with ribosome biogenesis, probably in an effort to arm their bactericidal mechanisms and survive. Of interest and in a similar way to Mφ, *in vitro* infection of PMNs with *Brucella* led to downregulated gene expression in key molecular pathways for PMNs physiology and function including phosphatidylinositol signaling, TNF signaling, and cellular senescence. Phosphatidylinositol signaling pathway plays an important role in membrane dynamics and trafficking, including proteins implicated in endosomal membranes and autophagosome assembly and activity (37, 38). Autophagy is closely related to the intracellular lifestyle of many pathogens, including *Brucella* (39). We hypothesize that the downregulation of several autophagy sensors and regulators belonging to phosphatidylinositol pathway further modulates the autophagic capacity of PMNs against *Brucella*. This may also explain the inability of *Brucella*-infected PMNs to form neutrophil extracellular traps (NETs) (17), an effector mechanism positively associated with the autophagy machinery (40). Downregulation of the cellular senescence pathway is in agreement with the reported premature death of *Brucella*-infected PMNs (17). Additionally, senescence has been associated with resistance to cell death (41). Moreover, it appears that perturbation of TNF signaling represents a common stealth strategy of *Brucella* to avoid both Mφ- and PMNs-induced inflammation further restricting cellular immunity (8).

Human brucellosis causes high clinical morbidity and protean clinical manifestations, mimicking many infectious and non-infectious diseases, as any organ can be affected. The definition and diagnosis of different disease types of human brucellosis, such as acute, chronic/relapsing, asymptomatic/subclinical, and cured, continues to be challenging making the therapeutic decision difficult in many cases. This is due to various factors including the non-specific and atypical clinical features, the slow growth rate of *Brucella* in blood cultures and the reduced sensitivity of the method for detecting chronic cases. Furthermore, laboratory diagnosis in people living in endemic regions, high-risk occupational groups and previously infected individuals, as well as cross-reactivity in some serological assays renders challenging the serodiagnosis of brucellosis (3, 4, 10, 12).

To investigate the impact of human brucellosis on host immunity and identify possible candidate markers of active disease and response to treatment, we next assessed the transcriptome profiling of PBMCs and PMNs isolated from newly diagnosed patients with acute brucellosis, before and three months after their successful treatment. We observed, both in PBMCs and PMNs, transcriptomic alterations related to major pathways of inflammation, supporting its role in infection overcome. PBMCs from patients successfully treated were characterized by the overexpression of genes critically involved in hypoxia (*HIF1A*) and IL-1 signaling, and the downregulation of genes implicated in oxidative phosphorylation, lymphocyte activation, and cytotoxicity. In line with these data, a recent experimental study has demonstrated that absence of HIF-1α renders mice susceptible to *Brucella* infection, while HIF-1α reduces oxidative phosphorylation and increases glycolysis leading to inflammasome activation and IL-1β release in infected macrophages (42).

Treatment of brucellosis led to increased expression of several genes related to autophagy machinery in PMNs, including *DDIT4/REDD1* encoding a key regulator of autophagy-mediated NET formation (43). It seems that after clearance of infection, PMNs restored critical functions impaired by *Brucella*, such as autophagy. However, they did not acquire a proinflammatory phenotype as indicated by the downregulated expression in genes related NOD-like receptor signaling and cytokine-cytokine interaction pathways.

Comparison of the transcriptomic profiling of *ex vivo* PMNs after successful completion of treatment versus that of *in vitro Brucella*-infected PMNs, showed a substantial overlap, as 59% of the *ex vivo* identified DEGs were commonly regulated in both datasets. However, data derived from *in vitro* infected cells under “controlled” laboratory conditions cannot simulate completely the complex cellular interactions that occur upon human infection, or the possible differences in the kinetics by which certain processes unfold *in vitro* versus *ex vivo*.

Of note, this study identified a common set of 24 genes that were differentially expressed both in PMNs and PBMCs suggesting candidate molecular diagnostic/prognostic targets for human brucellosis. Among them, type II IFN pathway, which is the major driver of Th1 immunity against *Brucella* (5), appears to be induced in active disease and attenuated after treatment. Indeed, using patients’ sera, we confirmed at the protein level, that IFN-γ and other Th1 cytokines, such as IL-1β and IL-6, were increased during active disease and significantly diminished in cured, non-relapsed patients, whereas the levels of IFN-α, which belongs to type I IFN family, did not show significant changes. Collectively, these results confirmed past studies highlighting the significant role of a robust Th1 response to tackle acute infection and brucellosis-acquired cellular anergy of chronic disease (44–46).

In conclusion, this study provides an integrated transcriptome landscape of immune cells signature in human brucellosis suggesting candidate molecular pathways and targets for active disease and response to treatment. Based on these data, future validation and mechanistic studies may further decipher the pathogenesis of this ancient and continuously re-emerging zoonotic disease (1, 2, 47).

## Data availability statement

The datasets presented in this study can be found in online repositories. The name of the repository and accession numbers can be found below: NCBI Sequence Read Archive; PRJNA812759 and PRJNA812762.

## Ethics statement

The studies involving human participants were reviewed and approved by Local Scientific and Ethics Committee of the University Hospital of Alexandroupolis, Greece (Approval Number #1195/19-12-2017). The patients/participants provided their written informed consent to participate in this study.

## Author contributions

IM: Conceptualization, Funding acquisition, Visualization, Writing - original draft, Writing – review and editing. AC: Formal analysis, Investigation, Writing - review and editing. GD: Investigation, Data curation, Formal analysis, Writing - review and editing. CI: Data curation, Investigation. MN: Investigation, Validation. AT: Data curation. TK, CA, NS, SG and MP: Investigation. GL: Methodology, Validation. SM: Validation. ML, AD: Formal analysis. PS: Formal analysis, Methodology. UC: Methodology, Writing - review and editing. BW, KR: Writing - review and editing. PSk: Conceptualization, Funding acquisition, Project administration, Supervision, Writing - original draft, Writing - review and editing. All authors contributed to manuscript revision, read, and approved the submitted version.

## Funding

This study was supported by the German Federal Ministry for Education and Research (BMBF) and Greek General Secretariat for Research and Technology (GSRT), Greek-German Bilateral Research & Innovation Programme BRIDGING, grants MIS 5030062, 01EI1703A and 01EI1703B, and by the GSRT, Research & Innovation Programme CYTONET, grant MIS 5048548.

## Acknowledgments

We thank Prof. Triantafyllos Chavakis for his support and advice, and Dr. Apostolos Vasileiou for his technical and administrative assistance.

## Conflict of interest

Authors NS and GL were employed by the company P. Zafiropoulos S.A.

The remaining authors declare that the research was conducted in the absence of any commercial or financial relationships that could be construed as a potential conflict of interest.

## Publisher’s note

All claims expressed in this article are solely those of the authors and do not necessarily represent those of their affiliated organizations, or those of the publisher, the editors and the reviewers. Any product that may be evaluated in this article, or claim that may be made by its manufacturer, is not guaranteed or endorsed by the publisher.
