# Supplementary material for: A gene expression map of host immune response in human brucellosis
Source: Front Immunol. 2022 Aug 1;13:951232. doi: 10.3389/fimmu.2022.951232 (PMC9376622; doi:10.3389/fimmu.2022.951232)
Supplement: Supplementary file 2 [file DataSheet_2.pdf]

frontiers

Supplementary Material

Supplementary Table 1. Diagnostic laboratory findings at relapse and remission of patients with chronic relapsing brucellosis (24 males/1 female, mean age 60.6±6.9 years).

#Patient	Blood culture		Wright's SAT (positive ≥1:160)		Coombs' anti-*Brucella* SAT (positive ≥1:320)		Complement Fixation test (positive ≥1:16)	
	**Rel**	**Rem**	**Rel**	**Rem**	**Rel**	**Rem**	**Rel**	**Rem**
**CRB1**	pos	neg	160	160	640	640	32	32
**CRB2**	neg	neg	320	160	>1280	640	32	16
**CRB3**	pos	neg	160	80	640	320	64	16
**CRB4**	pos	neg	80	80	320	320	64	32
**CRB5**	neg	neg	320	320	>1280	>1280	256	64
**CRB6**	neg	neg	160	160	640	640	128	64
**CRB7**	pos	neg	160	160	1280	320	128	16
**CRB8**	neg	neg	320	640	>1280	>1280	32	16
**CRB9**	pos	neg	160	160	640	640	128	32
**CRB10**	neg	neg	160	80	160	160	64	16
**CRB11**	neg	neg	80	160	1280	640	128	32
**CRB12**	neg	neg	160	80	320	320	16	32
**CRB13**	pos	neg	80	40	320	160	256	32
**CRB14**	neg	neg	80	160	320	640	128	64
**CRB15**	neg	neg	640	160	>1280	640	64	64
**CRB16**	neg	neg	320	160	320	160	128	64
**CRB17**	neg	neg	160	80	640	160	256	32
**CRB18**	neg	neg	320	640	>1280	>1280	32	16
**CRB19**	pos	neg	160	160	640	640	128	32
**CRB20**	neg	neg	160	80	160	160	64	16
**CRB21**	neg	neg	80	160	1280	640	128	32
**CRB22**	neg	neg	160	80	320	320	16	32
**CRB23**	neg	neg	80	160	320	640	128	64
**CRB24**	neg	neg	640	160	>1280	640	64	64
**CRB25**	pos	neg	640	320	1280	>1280	>256	32

CRB; chronic relapsing brucellosis, rel; relapse, rem; remission, pos; positive, neg; negative

SAT; serum agglutination test. Results of serological tests are expressed as reciprocal titers.

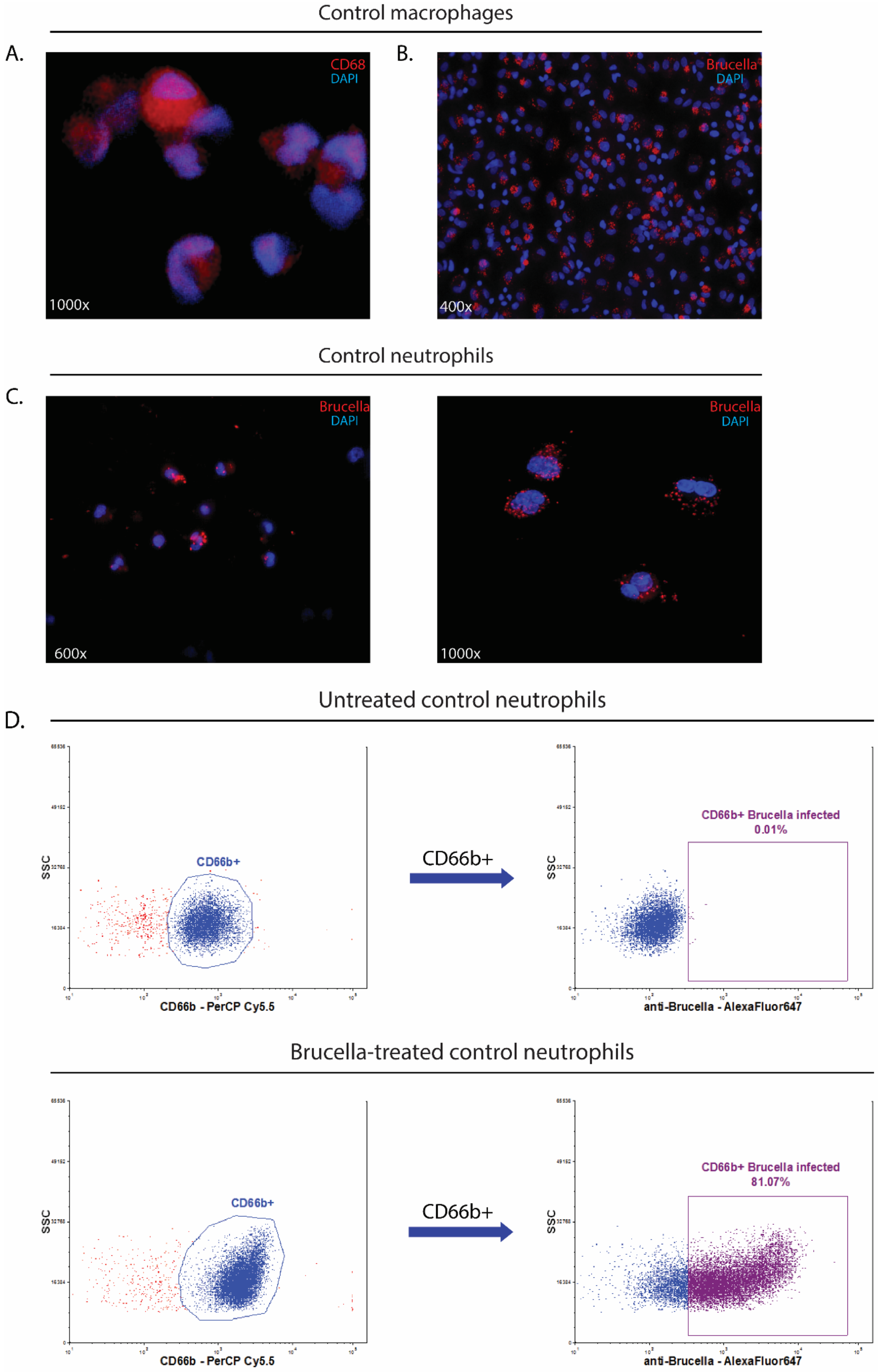
Control macrophages
A.
CD68
DAPI
1000x
B.
Brucella
DAPI
400x
Control neutrophils
C.
Brucella
DAPI
600x
Brucella
DAPI
1000x
Untreated control neutrophils
D.
SSC
CD66b+
CD66b - PerCP Cy5.5
CD66b+
CD66b+ Brucella infected
0.01%
anti-Brucella - AlexaFluor647
Brucella-treated control neutrophils
CD66b+
CD66b - PerCP Cy5.5
CD66b+
CD66b+ Brucella infected
81.07%
anti-Brucella - AlexaFluor647

Supplementary Figure 1. Differentiation status of human macrophages and phagocytosis of *Brucella* by human macrophages and neutrophils. (A) Monocyte-to-macrophage differentiation was verified by immunofluorescence staining (Red: Mouse monoclonal anti-CD68, Blue: DAPI) using a fluorescence microscope, 100x lens. Macrophages and neutrophils from healthy donors (control) were treated *in vitro* with opsonized *Brucella* for 30 min, and phagocytosis was assessed by cell immunofluorescence (Red: Mouse monoclonal anti-*Brucella*, Blue: DAPI): (B) Control macrophages. Visualization was performed using a fluorescence microscope, 40x lens. (C) Control neutrophils. Left panel: Visualization was performed using a fluorescence microscope, 60 x lens. Right panel: Visualization was performed using a confocal microscope, 100 x lens. (D) Flow cytometric analysis in control neutrophils. CD66b positive cells were examined for the presence of *Brucella*, upon staining with a mouse monoclonal anti-*Brucella* antibody detected with a rabbit anti-mouse Alexa Fluor 647. Untreated neutrophils from a healthy donor (Untreated control neutrophils) were served as negative control. For (A)-(D), representative figures are shown.

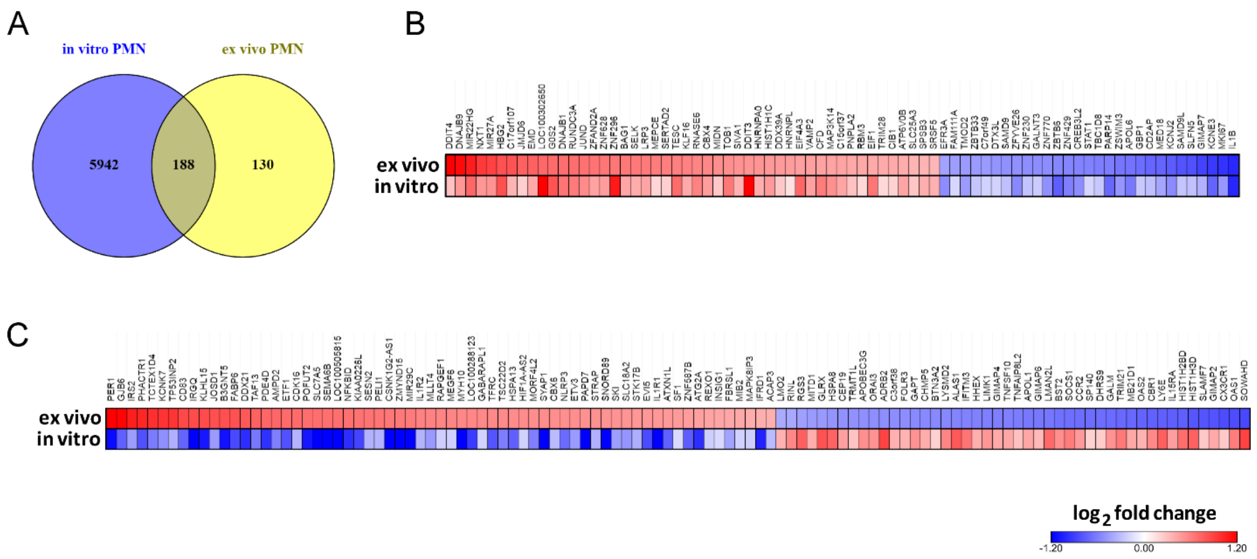

Supplementary Figure 2. Comparison of transcriptomic profile of *in vitro* infected PMNs and PMNs from patients initially diagnosed with active brucellosis, after successful completion of their treatment. (A) Venn diagram showing that 188 genes are commonly regulated between the two datasets. (B) and (C) Heatmaps showing the common differentially expressed genes (DEGs), characterized by similar (B, 77 DEGs) or opposite pattern of differential expression (C, 111 DEGs). FDR < 0.1 was used as threshold for both datasets.
